# Men’s comfort in distributing or receiving HIV self-test kits from close male social network members in Dar Es Salaam, Tanzania: baseline results from the STEP project

**DOI:** 10.1186/s12889-021-11806-5

**Published:** 2021-09-24

**Authors:** Joseph K. B. Matovu, Gaspar Mbita, Akeen Hamilton, Frank Mhando, Wynton M. Sims, Noah Thompson, Albert N. Komba, Jackson Lija, Jiajia Zhang, Thomas van den Akker, Dustin T. Duncan, Augustine T. Choko, Donaldson F. Conserve

**Affiliations:** 1 Makerere University School of Public Health, Kampala, Uganda; 2 Busitema University Faculty of Health Sciences, Mbale, Uganda; 3 Vrije Universiteit, Amsterdam, Netherlands; 4 Jhpiego Tanzania, An Affiliate of Johns Hopkins University, Dar-es-Salaam, Tanzania; 5 Arnold School of Public Health, University of South Carolina, Columbia, SC USA; 6 University of Dar es Salaam, Dar es Salaam, Tanzania; 7 Stellenbosch University, Business School, Stellenbosch, South Africa; 8 School of Public Health, The University of Alabama at Birmingham, Birmingham, AL USA; 9 National AIDS Control Program, Ministry of Health Community Development, Gender, Elderly, and Children, Dodoma, Tanzania; 10 Columbia University Mailman School of Public Health, New York, USA; 11 Malawi-Liverpool Wellcome Trust Clinical Research Programme, Blantyre, Malawi; 12 Department of Prevention and Community Health, Milken Institute School of Public Health, The George Washington University, Washington, DC, USA

**Keywords:** HIV, Men, HIV self-testing, Social networks, Tanzania

## Abstract

**Background:**

A variety of strategies have been used to reach men with HIV self-testing services, including social network-based HIV self-test kits distribution. However, few studies have assessed men’s comfort to distribute to or receive HIV self-test kits from close male friends within the same social network. In this study, we assessed men’s comfort to distribute to and/or receive HIV self-test kits from close male friends and associated factors among men who socialize in networks locally referred to as “camps” in Tanzania.

**Methods:**

Data are from the baseline survey of a cluster-randomized controlled trial conducted in June 2019 with 18 social networks or “camps” in Dar es Salaam, Tanzania. Participants were 18-year-old or older male camp members who were HIV-negative at the time of enrolment. We used the Generalized Estimating Equations (GEE) to assess factors associated with being comfortable to distribute to and/or receive HIV self-test kits from close male members within one’s social network.

**Results:**

Of 505 participants, 67.9% (*n* = 342) reported being comfortable to distribute to while 68.2% (*n* = 344) were comfortable to receive HIV self-test kits from their close male friends. Ever having heard about HIV self-testing (Adjusted Prevalence Ratio (Adj. PR): 1.6; 95% Confidence Interval [CI]: 1.3, 1.9), willingness to self-test for HIV in front of a sexual partner (Adj. PR: 3.0; 95%CI: 1.5, 6.1) and exposure to peer-led HIV self-testing education and promotion (Adj. PR: 1.4; 95%CI: 1.2, 1.7) were significantly associated with being comfortable to distribute HIV self-test kits to close male members within one’s social network. Similar results were observed for being comfortable to receive HIV self-test kits from a close male friend within one’s social network.

**Conclusions:**

Overall, these findings suggest that distribution of HIV self-test kits through close male friends could improve the proportion of men reached with HIV self-testing services and improve HIV testing rates in this population where uptake remains low. However, additional promotional strategies such as peer-led HIV self-testing education are needed to raise awareness and increase the proportion of men who are comfortable to receive and/or distribute HIV self-testing kits.

## Background

In many settings, HIV testing rates and linkage to HIV care among people living with HIV (PLWH) remain much lower among men than women [1]. Consequently, a report from the Joint United Nations Programme (UNAIDS) on HIV/AIDS referred to men and boys as the ‘blind spot’ in the HIV prevention response [2]. Citing survey and program reports from 25 sub-Saharan African countries, the report indicated that in nearly half (12) of these countries, men living with HIV were leas likely to know their HIV status and knowledge of HIV status among men living with HIV was about half the rate found among women [2]. Thus, while HIV testing uptake among men has generally improved in recent years [3–5], it remains below the first “95” of UNAIDS 95–95-95 targets [6]. Hegemonic masculinity norms [7], coupled with high levels of stigma and discrimination around HIV testing [8], continue to keep some men away from getting tested for HIV, coming to terms with their HIV-positive status, taking instructions from nurses, and engaging in health enabling behaviors. These dynamics contribute to poorer HIV testing uptake and treatment outcomes for men compared to women in high prevalence settings, calling for innovative approaches to reach men with HIV testing and linkage to HIV care services.

Evidence from prior studies has demonstrated that HIV self-testing is an acceptable and efficacious to increase uptake of testing services and facilitate linkages to care, especially among persons who are at high risk of HIV infection [9–11]. Several approaches have been employed to reach men with HIV self-testing services, including household visits by community HIV care providers [12], female-delivered HIV self-testing kits [4, 13–15], delivery through existing social networks [16], and distribution through the internet; particularly among men who have sex with men [17–19]. Using a social network strategy to distribute HIV self-test kits to African American and Latino men who have sex with men in Alameda County, California, in the United States of America, Lightfoot et al. [17] found that individuals reached through a peer-based HIV self-testing strategy were significantly more likely to have never tested for HIV than men who have sex with men reached through community-based HIV testing programs. Similarly, Lippman et al. [18] found that network distribution of HIV self-test kits not only reached men who have sex with men who were testing for the first time but also increased the frequency of HIV testing from 37.8 to 84.5% after the introduction of HIV self-test kits in South Africa. MacGowan et al. [20] reported 34 (1.6%) infections among 2152 social network members who received HIV self-test kits from study participants in the United States of America. Tun et al. [19] reported 100% linkage to HIV care among men who have sex with men who were identified through an intervention that involved HIV self-testing through key opinion leaders in Nigeria.

Collectively, these studies indicate that men are willing to use HIV self-test kits to test for HIV [9, 11, 21], with additional evidence suggesting that men are willing to self-test for HIV if they receive HIV self-test kits from their friends or sexual partners [16, 22]. Fleming and other scholars have argued that use of gender-transformative approaches in which men are engaged to reach fellow men with HIV prevention interventions can create safety nets within which men can discuss issues that affect their own health, including HIV testing [6, 23]. Studies among existing social networks of heterosexual men from the general population in Tanzania have found that men already engage in HIV testing conversations with friends in their social networks [21, 24] and that discussing HIV testing with a sexual partner and having been encouraged to test for HIV by a close friend were associated with higher odds of being willing to self-test among men [9, 21]. While these studies have provided preliminary evidence for the potential to leverage men’s social networks to deliver HIV self-test kits, no research has been conducted to examine heterosexual men’s willingness to distribute HIV self-test kits to fellow men or receive HIV self-test kits from fellow men in Tanzania, where 51% of men living with HIV were not aware of their HIV status in 2017 [25].

In 2018, there were 72,000 new cases of HIV infection among adults aged 15 years and older in in Tanzania [25]. The population which is significantly affected by HIV in Tanzania include people who inject drugs, mobile populations, young people, and men who have sex with men [26]. Mobile male populations, including truck drivers, plantation workers, and fishermen have also been found to be at increased risk of HIV infection [24]. In response, the government of Tanzania developed national HIV prevention strategies, including HIV self-testing, [27] and launched a national Test and Treat campaign focused on increasing HIV testing among men [28]. However, in Tanzania as well as elsewhere, the majority of male peer-delivered HIV self-testing studies have been conducted with men who have sex with men [18, 29–31]. Thus, there is limited evidence on how best to reach heterosexual men with HIV self-testing through male peer-delivered approaches.

In this study, we use baseline data from the Tanzania STEP (Self-Testing Education and Promotion) project [32] to investigate factors associated with men’s comfort to distribute or receive HIV self-test kits from close male friends among heterosexual men who socialize in networks locally referred to as “camps” in Dar es Salaam, Tanzania in order to inform implementation of social network-based HIV self-test kits distribution among heterosexual men.

## Methods

### STEP project overview

The STEP project was a five-year study funded by the University of North Carolina (UNC) Center for AIDS Research and the National Institute of Mental Health that was developed as part of a collaboration between the University of South Carolina, Jhpiego Tanzania, Tanzania Commission for AIDS (TACAIDS), UNC at Chapel Hill, Muhimbili University of Health and Allied University, EngenderHealth, and the National AIDS Control Programme of the Tanzanian Ministry of Health, Community Development, Gender, Elderly and Children (MoHCDGEC).

### Study site and population

The study was conducted among heterosexual young (18–24 years) and adult men (25 years or older) recruited from social networks or “camps” in Manzese and Tandale wards of Dar es Salaam, Tanzania. Camps have been described in previous publications [33, 34]. In brief, camps are social groups, comprising mostly young men, that have been in existence for over a decade [33]. Camps were first identified by our research team between 2007 and 2008 during a neighborhood mapping exercise to locate networks of men for a community-based intervention that leveraged men’s social networks to promote HIV and gender-based violence prevention [33, 35]. Approximately 500 camps were identified and 60 of these camps were included in a larger cluster randomized controlled trial (cRCT) conducted between 2013 and 2017 [36]. Each camp is defined by a unique name that is occasionally written in a geographical space, where camp members meet and socialize [33].

To be eligible for the STEP project, camps were required to have participated as one of 30 control-group camps in the larger cRCT and have between 10 and 70 members. To verify that the camps were still active at the time the STEP project began, the study team visited the camps between August and September 2018 to collect a roster of active camp members. A total of 18 camps were selected for the STEP project and the roster served as the sampling frame to confirm participants were active camp members. Eleven (11) camps in Manzese and seven (7) in Tandale wards were eligible for randomization. Constrained randomization conducted by author JZ with respect to camp size and geographical location was implemented through the “Efficient Design and Analysis of Cluster Randomized Trials” (“cvcrand”) package in R. Two groups with five or six camps each in Manzese ward and two groups with three or four camps each in Tandale ward were randomized into either the intervention group (*N* = 9) or the control group (*N =* 9).

### Data collection

Data are from the baseline survey that was conducted with members from the selected 18 camps. Members were recruited in June 2019 if they were male, aged 18 years or older, a camp member for at least 3 months, and self-reported to be HIV-negative at enrollment. Written informed consent was obtained from all participants prior to data collection. Following the informed consent, participants were administered a questionnaire by a research assistant at the study site, two pop-up tents placed near the camp sites. The survey was uploaded on Qualtrics and administered in the Kiswahili language via a Samsung Tablet. Data were collected from June 10–30, 2019. A total of 508 participants were screened; three participants did not meet eligibility criteria and 505 consented to participate into the study. During data collection, participants were asked if they had ever heard of HIV self-testing. All participants received an explanation of HIV self-testing before being asked about their comfort level with distributing to or receiving an HIV self-test kit from a close male friend. The baseline survey covered broad topics including demographics, HIV testing history, knowledge of HIV self-testing, and prior HIV self-test use. Participants received compensation equivalent to $4.50 USD.

### Outcome measures

Outcome measures used in the analyses were whether a participant was comfortable (1) distributing HIV self-test kits to close male friends in their social network, or (2) receiving HIV self-test kits from close male friends in their social network. Outcome variables were measured with the following no/yes questions: (1) *Would you feel comfortable distributing HIV self-test kits to your close male friends*? and (2) *Would you feel comfortable receiving HIV self-test kits from your close male friends?*

### Demographic and HIV testing measures

Demographic measures included age, education, and marital status. Age was categorized into three groups (18–24 years, 25–34 years, and 35 years or older). Education was classified as no formal education, primary education, or secondary education or more. Marital status was categorized as single, married/cohabiting, or divorced/widowed. Whether a participant was in a camp that participated in the peer-led HIV self-testing promotion intervention was also recorded. Participants were asked if they had ever been tested for HIV and if they had ever heard of HIV self-testing; these variables were categorized as no/yes. Based on previous findings showing the influence of social networks on willingness to self-test for HIV [9], participants were asked about their comfort with receiving an HIV self-testing kit from a close friend and/or distributing an HIV self-testing kit to them. Participants were also asked if their close male friend had ever encouraged them to self-test for HIV; this variable was categorized as no/yes. Regarding sexual network, participants were asked if they had ever discussed HIV self-testing with a sexual partner and if they were willing to use an HIV self-test kit in front of a sexual partner; these variables were categorized as no/yes.

### Statistical analysis

Data across the intervention and control arms were pooled and analyzed using STATA software version 15.0 (Stata Statistical Software: Release 15. 2017. College Station, TX: StataCorp LLC.). Following data cleaning and checking for consistency and completeness, descriptive statistics were summarized. Measures of central tendency (median) and respective measures of dispersion (interquartile range) were used to summarize continuous variables (i.e., age), which provide the evidence for categorization. Frequency and percentages were used to summarize categorical variables. The distribution of independent variables with the two main outcome variables (comfortable distributing HIV self-test kits to a close male friend and comfortable receiving HIV self-test kits from a close male friend) were investigated with a Chi-square test. Multiple poisson regression model was used to model the relationship between the main outcome variables and the independent variables where the clustering effect was included by the random intercept. The model was estimated through the Generalized Estimating Equations (GEE) approach. All independent variables with a significance association (*p* < 0.05) with the outcome variable in the crude analysis were incorporated into the multivariable regression model. Crude and adjusted prevalence rate and respective 95% confidence intervals were used to interpret the magnitude of association; the criterion for statistical significance was set at a *p*-value of < 0.05.

## Results

### Participants’ characteristics

Table 1 shows the background characteristics of the participants. The median age is 27 years (interquartile range = 22, 34) with 40% in age 18–24 and 40% in age 25–34. Ninety-six percent of the participants (*n* = 485) had primary or higher education. Majority of participants (60.2%) were single. Nearly nine of every ten participants (90.3%, *n* = 456) had ever tested for HIV while 42.6% (*n* = 215) had ever heard about HIV self-test kits. Thirty-eight percent (*n* = 190) reported that their close male friends had encouraged them to self-test for HIV. Eighty-three percent (*n* = 353) reported that they were willing to use HIV self-test kits in front of their sexual partners. Of the 505 participants surveyed, 50.1% (*n* = 253) from the 9 intervention camps received peer-led HIV self-test education and promotion for one month prior to participating in the baseline survey.

**Table 1:** Background characteristics and proportion of study participants who were comfortable to distribute to/receive HIV self-test kits from close male friends

Characteristic	Background Characteristics of study participants (*N* = 505)	Proportion of study participants who were comfortable to distribute HIV self-test kits to close male friends (*N* = 342)		Proportion of study participants who were comfortable to receive HIV self-test from close male friends (*N =* 344)	
	n (%)	n (%)	*p-*value*	n (%)	*p-*value*
**Age**					0.016
18–24	200 (38.6)	127 (63.5)	0.025	127 (63.5)	
25–34	201 (39.8)	134 (66.7)		135 (67.2)	
35+	104 (20.6)	81 (78.6)		82 (79.6)	
**Education level**					0.233
No formal education	20 (4.0)	14 (73.7)		16 (84.2)	
Primary education	247 (48.9)	168 (68.0)	0.843	171 (69.2)	
Secondary & above	238 (47.1)	160 (67.2)		157 (66.0)	
**Marital Status**					0.368
Single	304 (60.2)	199 (65.7)		200 (66.0)	
Married/Cohabiting	183 (36.2)	131 (71.6)	0.399	132 (72.1)	
Divorced/ Widowed	18 (3.6)	18 (66.7)		12 (66.7)	
**Ever tested for HIV**					0.938
No	49 (9.7)	32 (66.7)		33 (68.8)	
Yes	456 (90.3)	310 (68.0)	0.853	311 (68.2)	
**Ever heard of HIV self-testing**					< 0.001
No	290 (57.4)	133 (46.0)	< 0.001	133 (46.0)	
Yes	215 (42.6)	209 (97.2		211(98.1)	
**Ever been encouraged by close male friend to self-test for HIV**					< 0.001
No	314 (62.3)	159 (50.6)	< 0.001	158 (50.3)	
Yes	190 (37.7)	183 (96.3)		186 (98.0)	
**Willingness to use HIV self-test kits in front of sexual partner**					< 0.001
No	71 (16.8)	11 (15.5)	< 0.001	11 (15.5)	
Yes	353 (83.2)	276 (78.2)		280 (79.3)	
**Exposure to HIV self-testing promotion**					< 0.001
No	252 (49.9)	120 (47.6)	< 0.001	119 (47.2)	
Yes	253 (50.1)	222 (88.1)		225 (89.3)	
**Comfortable distributing HIV self-test kits to close male friends**					
No	162 (32.1)				
Yes	342 (67.9)				
**Comfortable receiving HIV self-test kits from close male friends**					
No	160 (31.8)				
Yes	344 (68.2)				

*p-values were derived from the Chi-Square test

### Being comfortable to distribute HIV self-test kits to close male friends

Table 1 shows the proportion of study participants who were comfortable to distribute to close male friends. Overall, 67.9% (*n* = 342) of the participants reported that they would be comfortable to distribute HIV self-test kits to their close male friends. Men aged 35 years or older (78.6%, *n* = 81), those that had ever heard about HIV self-testing (97.2%, *n* = 209) and those that were encouraged to self-test for HIV by their male friends (96.3%, *n* = 183) were significantly more likely to report that they would be comfortable to distribute HIV self-test kits to their close male friends than their counterparts (*P* < 0.001). In addition, participants who reported that they were willing to use the kits in front of their sexual partners (78.2%, *n* = 276) and those that received peer-led HIV self-testing education and promotion (88.1%, *n* = 222) were also significantly more likely to report that they would be comfortable to distribute HIV self-test kits to their close male friends than their counterparts (*P* < 0.001).

### Being comfortable to receive HIV self-test kits from close male friends

Table 1 also shows the proportion of study participants who were comfortable to receive HIV self-test kits from close male friends. Overall, 68.2% (*n* = 344) of the participants reported that they would be comfortable to receive HIV self-test kits from their close male friends. Individuals aged 35 years or older (79.6%, *n* = 82), those that had ever heard about HIV self-testing (98.1%, *n* = 211), those that had been encouraged to self-test for HIV by their male friends (98%, *n* = 186), and those that were willing to use the HIV self-test kit in front of their sexual partners (79.3%, *n* = 280) were also significantly more likely to report that they would be comfortable receiving HIV self-test kits from their close male friends than their counterparts (*P* < 0.001). As expected, participants who were exposed to peer-led HIV self-testing education and promotion were also significantly more likely to report that they were willing to receive HIV self-test kits from their close male friends than their control-arm counterparts (*P* < 0.001).

Table 2 shows the crude and adjusted prevalence ratios associated with being comfortable to distribute HIV self-test kits to a close male friend. After controlling for other factors and taking clustering into account, having ever heard about HIV self-testing (adjusted Prevalence Ratio [Adj. PR] = 1.6; 95%CI: 1.3, 1.9), male friend’s influence on HIV self-testing (Adj. PR: 1.5; 95%CI: 1.2, 1.8), willing to use HIV self-test kits in front of a sexual partner (Adj. PR: 3.0; 95%CI: 1.5, 6.1) and exposure to peer-led HIV self-testing education and promotion (Adj. PR: 1.4; 95%CI: 1.2, 1.7) remained significant factors associated with being comfortable to distribute HIV self-test kits to a close male friend.

**Table 2:** Factors associated with being comfortable to distribute HIV self-test kits from a close male friend

	Crude Prevalence Ratio		Adjusted Prevalence Ratio	
**Variable**	**PR (95% CI)**	*p-value	**PR (95% CI)**	*p-value
**Age categories**				
18–24	1		1	
25–34	1.0 (0.8, 1.3)	0.697	1.1 (0.9, 1.2)	0.333
35+	1.2 (0.9, 1.6)	0.118	1.3 (1.1, 1.5)	0.030
**Education level**				
No formal education	1		1	
Primary education	0.9 (0.7, 1.1)	0.506	1.0 (0.7, 1.4)	0.993
Secondary and above	0.9 (0.7, 1.2)	0.505	1.1 (0.8, 1.5)	0.634
**Marital Status**				
Single	1		1	
Married/ Cohabiting	1.1 (0.9, 1.3)	0.372	0.9 (0.8, 1.0)	0.071
Divorced/ Widowed	1.0 (0.7, 1.5)	0.940	0.7 (0.4, 1.0)	0.059
**Ever tested for HIV**				
No	1		1	
Yes	1.0 (0.9, 1.2)	0.811	0.9 (0.8, 1.1)	0.464
**Ever heard about HIV self-testing**				
No	1		1	
Yes	2.1 (1.4, 3.1)	< 0.001	1.6 (1.3, 1.9)	< 0.001
**Male friend influence on HIV self-testing**				< 0.001
No	1	0.001	1	
Yes	1.9 (1.3, 2.8)		1.5 (1.2, 1.8)	
**Willing to use HIV self-test kits in front of a sexual partner**				0.002
No	1		1	
Yes	5.0 (2.1, 12.2)	< 0.001	3.0 (1.5, 6.0)	
**Exposure to HIV self-testing promotion**				< 0.001
No	1	< 0.001	1	
Yes	1.9 (1.4, 2.4)		1.4 (1.2, 1.7)	

*p-values were derived from the Wald test

Table 3 shows the crude and adjusted prevalence ratios associated with being comfortable to receive HIV self-test kits from a close male friend. After controlling for other factors and taking clustering into account, having ever heard about HIV self-testing (Adj. PR: 1.5; 95%CI: 1.3, 1.8), male friend’s influence on HIV self-testing (Adj. PR: 1.5; 95%CI: 1.3, 1.9), willingness to use HIV self-testing kits in front of a sexual partner (Adj. PR: 3.0; 95%CI: 1.5, 6.0) and exposure to peer-led HIV self-testing education and promotion (Adj. PR: 1.5; 95%CI: 1.2, 1.8) were significantly associated with being comfortable to receive HIVST kits from a close male friend.

**Table 3:** Factors associated with being comfortable to receive HIV self-test kits from a close male friend

	Crude Prevalence Ratio		Adjusted Prevalence Ratio	
**Variable**	**PR (95% CI)**	*p-value	**PR (95% CI)**	*p-value
**Age categories**				
18–24	1		1	
25–34	1.1 (0.8, 1.4)	0.667	1.1 (0.9, 1.2)	0.349
35+	1.3 (1.0, 1.6)	0.105	1.2 (1.0, 1.5)	0.051
**Education level**				
No formal education	1		1	
Primary education	0.8 (0.7, 1.0)	0.089	0.9 (0.7, 1.3)	0.646
Secondary and above	0.8 (0.6, 1.0)	0.079	0.9 (0.7, 1.3)	0.717
**Marital Status**				
Single	1		1	
Married/ Cohabiting	1.1 (0.9, 1.3)	0.341	0.9 (0.8, 0.9)	0.027
Divorced/ Widowed	1.0 (0.6, 1.6)	0.968	0.6 (0.4, 0.9)	0.048
**Ever tested for HIV**				
No	1		1	
Yes	1.0 (0.8, 1.2)	0.921	0.9 (0.8, 1.1)	0.382
**Ever heard about HIV self-testing**				
No	1		1	
Yes	2.1 (1.5, 3.1)	< 0.001	1.5 (1.3, 1.9)	< 0.001
**Male friend influence on HIV self-testing**				< 0.001
No	1	0.001	1	
Yes	1.9 (1.3, 2.9)		1.5 (1.3, 1.9)	
**Willing to use HIV self-test kits in front of a sexual partner**				0.001
No	1		1	
Yes	5.1 (2.1, 12.2)	< 0.001	3.0 (1.5, 6.0)	
**Exposure to HIV self-testing promotion**				< 0.001
No	1	< 0.001	1	
Yes	1.9 (1.4, 2.5)		1.4 (1.3, 1.8)	

*p-values were derived from the Wald test

## Discussion

Our results from the baseline study of the STEP Project showed a high level of comfort among men with distributing to and/or receiving HIV self-test kits from close male friends: 68% of participants indicated that they would be willing to distribute to and receive HIV self-test kits from their close male friends. These findings suggest the potential for peer-led HIV self-testing to reach men through fellow social network members and improve HIV testing rates among men who have never tested for HIV as well as among HIV-negative repeat HIV testers. The need for repeat HIV testing is particularly important because, compared to prior studies documenting low HIV testing rates among men in Tanzania [24, 37], most of the men in this study had been tested for HIV due to the ongoing national Test and Treat campaign focused on increasing HIV testing rates among men [28]. Formative qualitative research revealed that men perceived HIV self-testing as a facilitator for repeat HIV testing among HIV-negative testers because testing at home would save time compared to the commute and long queues associated with facility-based HIV testing [32].

Although we found that a high proportion (68%) of men were willing to distribute HIV self-test kits to their friends, another study conducted among men who have sex with men in the U.S. found a much higher proportion, with at least 90% of men who have sex with men reporting they would be comfortable to distribute HIV self-test kits within their social networks [30]. The lower proportion of men in our study who reported being comfortable to distribute an HIV self-test kit to their social network peers may be explained by the lack of familiarity with HIV self-testing for some of the men. This is supported by the fact that among men who had received peer-led HIV self-testing education and promotion, a much higher proportion (88%) of them reported being willing to distribute an HIV self-test kit to their peers compared to only 48% of men who had not been exposed to peer-led HIV self-testing education and promotion. These findings are supported by past research demonstrating that prior engagement with peer educators was associated with willingness to use pre-exposure prophylaxis among male sex workers in Vietnam [38].

Peer educators have been engaged in promoting uptake of HIV self-testing in several prior studies. Chanda et al. used peer educators for an HIV self-test cluster randomized controlled trial [39]. In the study, peer educators served to either directly distribute HIV self-test kits or distribute an HIV self-test coupon for female sex workers to collect the HIV self-test kit at a certain distribution point [39]. The peer educators, who were current or former female sex workers, were recruited by the study staff and participated in a two-day training. Throughout the study, the peer educators met with participants a minimum of four times in order to provide general related health education, provide knowledge on how to use the kits, and conduct follow-up visits after the women had tested [40]. Peer educators proved to have a positive effect on HIV self-testing among this population, pointing to the possibility that they served to decrease participants’ concerns about HIV-related stigma [40]. Results from another study conducted to assess the effect of peer-based distribution of HIV self-test kits among fishermen in Buliisa, Uganda, found that 82% (*n* = 95) of the fishermen accepted to receive HIV self-test kits from their peers; of these, 29 (25.8%) had never tested for HIV while 42 (44.2%) had tested more than a year ago [16]. In this study, 19 peers were recruited from patients attending health services at a facility as well as from community members and trained in how to distribute HIV self-test kits. Each peer received up to five HIV self-test kits for distribution to eligible social network members (i.e. those aged 18 years and above and who had not recently tested for HIV).

Taken together, these studies demonstrate that network-based distribution of HIV self-test kits can improve HIV testing rates, particularly in hard-to-reach populations, including men. However, the majority of peer-led HIV self-test kits distribution studies have not assessed the willingness to distribute HIV self-test kits among the peers who are reached by peer educators. Engaging peers who are reached by trained peer educators provides an opportunity to not only assess the effect of receiving peer-led HIV self-testing promotion but also an opportunity to leverage their knowledge of and use of HIV self-test kits to reach additional social and sexual network members that originally trained peer educators may not be able to reach. For example, we found that men who reported that their close male friends had encouraged them to self-test for HIV were more comfortable with receiving an HIV self-test kit from a close male friend than their counterparts. In the event that a close male friend is not a peer educator but may know the peer educator, this close male friend can receive two to three HIV self-test kits from a peer educator and then distribute them among his other friends who may not be close to the peer educators. Qualitative findings from the same group of men revealed that men, who were not peer educators, engage in HIV testing conversations with their friends and were willing to distribute HIV self-test kits to their close friends who may not want to seek HIV testing at the clinic [21]. Thus, more research is needed to explore the feasibility of expanding peer-based HIV self-test distribution to peers who are not reached by the peer educators.

We also found that men who were willing to use an HIV self-test kit in front of a sexual partner were more likely to be comfortable to receive an HIV self-test kit from a close male friend than their counterparts. This finding supports the potential for men to receive multiple HIV self-test kits from a close friend instead of only a peer educator for them to use with their sexual partners. A close male friend compared to a peer educator may have more information about another friend’s risky sexual behavior. This knowledge of close friend’s risky sexual behavior played a role in men encouraging their friends to test for HIV in Tanzania [21] and can be leveraged to have men distribute HIV self-test kits to their friends to also test their sexual partners. To our knowledge, there are no network-based studies that have provided heterosexual men with multiple HIV self-test kits to use with their female sexual partners. Rather, the existing studies have focused only on providing women multiple HIV self-test kits to use with their male sexual partners [4, 5, 13, 15]. These studies have shown that providing women with HIV self-test kits to use with their male partners can help to identify men who are unaware of their positive HIV status and reduce HIV risk for the female partner [4, 15]. Similarly, if men receive multiple HIV self-test kits to use with their sexual partners, it can help them assess their female partner’s HIV status and potentially prevent HIV acquisition from female partners who may be unaware of their positive HIV status. This is particular important because an earlier study conducted among men in Tanzania found that the majority of them were not aware of their sexual partner’s HIV status and that men who had two or more sexual partners were significantly less likely to be aware of their partner’s HIV status [37]. In addition, men who use condoms inconsistently were less likely to be aware of their sexual partner’s HIV status [37]. Thus, men, especially those with multiple sexual partners, can benefit from receiving multiple HIV self-test kits and proper guidance on how to self-test with their sexual partners as an HIV prevention strategy.

Although our study has several strengths, there are a few limitations worth highlighting, including the fact that data originated from a cross-sectional survey and were not collected to assess the causal relationship between variables of interest. Given the hypothetical nature of the outcome variables examined, the responses may vary in future studies with more or fewer men willing to distribute to or receive HIV self-test kits from close male friends. In addition, these findings are not generalizable to other men in the country since they were collected among men who are members of social networks called camps. However, this is one of the first studies to reach men who were exposed to peer-led HIV self-testing education and promotion to assess their willingness to distribute to or receive HIV self-test kits from close male social network members. The findings support the potential for network-based HIV self-testing distribution strategies to reach men who may not be close friends with peer educators.

## Conclusion

Our study showed that nearly seven out of ten men were comfortable to distribute to and receive HIV self-test kits from their close male friends within a social network setting. Having ever heard about HIV self-testing, willingness to self-test in front of one’s sexual partner, and exposure to peer-led HIV self-testing education and promotion were significantly associated with being comfortable to distribute to or receive HIV self-test kits from close male friends within the social network. These findings suggest that distribution of HIV self-test kits through close male friends could improve the proportion of men reached with HIV self-testing services and improve HIV testing rates in this population where uptake of HIV testing among men remains low.

## Data Availability

The datasets used and/or analysed during the current study are available from the corresponding author on reasonable request.

## Abbreviations

AIDS: Acquired Immune Deficiency Syndrome
cRCT: Cluster Randomized Controlled Trial
FSW: Female Sex Worker
HIV: Human Immunodeficiency Virus
JHPIEGO: Johns Hopkins Program for International Education in Gynecology and Obstetrics
MoHCDGEC: Ministry of Health, Community Development, Gender, Elderly and Children
UNC: University of North Carolina
PEPFAR: President’s Emergency Plan for AIDS Relief
PR: Prevalence Rate
STEP: Self-Testing Education and Promotion
TACAIDS: Tanzania Commission for AIDS
US: United States
USAID: United States Agency for International Development
